# In an Unpredictable and Changing Environment: Intrapreneurial Self-Capital As a Key Resource for Life Satisfaction and Flourishing

**DOI:** 10.3389/fpsyg.2017.01819

**Published:** 2017-10-23

**Authors:** Annamaria Di Fabio, Letizia Palazzeschi, Ornella Bucci

**Affiliations:** Department of Education and Psychology, Psychology Section, University of Florence, Florence, Italy

**Keywords:** intrapreneurial self-capital (ISC), life satisfaction, flourishing, career construction, life construction, healthy business, healthy organization

## Abstract

The twenty-first century is characterized by an unpredictable and challenging work environment, and the Intrapreneurial Self-Capital (ISC) career and life construct can be seen as a core of individual intrapreneurial resources that enables people to cope with ongoing challenges, changes, and transitions founding innovative solutions when confronted with the constraints imposed by such an environment. The ISC is a challenging construct since it can enhance behavior and attitudes through specific training, unlike personality traits, which are considered substantially stable in the literature. Against this background, the present study examined the relationship between ISC and well-being (hedonic well-being and eudaimonic well-being) controlling for the effects of personality traits. The Big Five Questionnaire (BFQ), the Intrapreneurial Self-Capital Scale (ISCS), the Satisfaction With Life Scale (SWLS), and the Flourishing Scale (FS) were administered to 258 Italian workers. Hierarchical regression analyses showed that ISC explained a percentage of incremental variance beyond that explained by personality traits in relation to both life satisfaction and flourishing. These results indicate that ISC is a key resource for hedonic well-being and eudaimonic well-being and that it offers new research and intervention opportunities.

## Introduction

The twenty-first century has so far been characterized by continuous economic challenges, globalization, and instability in the labor market (Savickas, 2011; Guichard, 2013). Occupational prospects are becoming increasingly unpredictable, and it is even more difficult to predict defined and stable life trajectories (Blustein, 2011, 2013; Savickas, 2011; Duffy et al., 2016).

In the face of this unpredictable and challenging environment, organizations try to introduce more innovation (García-Goñi et al., 2007) and workers need to adapt continually to change (Di Fabio and Gori, 2016a). In the current ever-changing world of work (Di Fabio and Kenny, 2016a), workers require the resources to deal with change constructively (Di Fabio and Gori, 2016a).

They can then devise new ways of dealing with challenges and transitions successfully thereby also promoting their own well-being (Zelenski et al., 2008; Van den Heuvel et al., 2010; Di Fabio and Palazzeschi, 2015; Di Fabio and Bucci, 2016). People who see change as an opportunity to learn and grow are far more likely to respond positively to the challenges of postmodern society (Wanberg and Banas, 2000; Van den Heuvel et al., 2013; Di Fabio, 2014; Di Fabio and Gori, 2016a). The great benefit of intrapreneurial self-capital (ISC, Di Fabio, 2014), as a core of individual intrapreneurial resources, is that it can assist people cope with frequent changes and transitions by helping them come up with innovative solutions to problems.

### Intrapreneurial self-capital

At the same time, ISC represents a challenging career and life construct, a new scale to measure this construct (Di Fabio, 2014), and an intervention in terms of specific training to build and improve the construct (Di Fabio and Van Esbroeck, 2016). The ISC is “a higher order construct containing seven subconstructs: (1) core self-evaluation as positive judgment of oneself in terms of self-esteem, self-efficacy, locus of control, and absence of pessimism (Judge et al., 2003); (2) hardiness as resistance with its three dimensions: commitment, control, and challenge (Maddi, 1990); (3) creative self-efficacy as one's perception of one's ability to solve problems creatively (Tierney and Farmer, 2002); (4) resilience as the perceived ability to cope with adversity adaptively and to use adaptive strategies to deal with discomfort and adversity (Tugade and Fredrickson, 2004); (5) goal mastery as the perceived ability to continuously develop one's own skills (Midgley et al., 2000); (6) decisiveness as the perceived ability to make decisions timeously in any life context (Frost and Shows, 1993); and (7) vigilance as the careful searching for relevant information (Mann et al., 1997)” (Di Fabio, 2014, pp. 100–102). Empirical studies so far have shown a positive relationship between ISC and scholastic success, career self-efficacy, and employability and a negative relationship with difficulties in making career decisions. What makes ISC particularly interesting is that it can be enhanced through specific training (Di Fabio, 2014) unlike personality traits, which are generally regarded as being substantially stable (McCrae and Costa, 1987).

ISC can, in fact, be seen as an individual resource that workers can use to face the uncertainty of the twenty-first century world of work and that can promote “healthy organizations” within a primary prevention perspective (Hage et al., 2007; Di Fabio and Kenny, 2015). In the postmodern era, the role of a positive work environment in enhancing the health and well-being of workers is widely recognized (Sparks et al., 2001) and calls for an organizational positive psychology approach in an organization (Tetrick and Peiró, 2012; Snyder et al., 2014; Di Fabio and Kenny, 2015; Di Fabio and Gori, 2016a,b) centered on fostering positive individual resources to promote employee well-being and flourishing, resilient workers (Di Fabio and Saklofske, 2014b; Di Fabio and Kenny, 2015; Di Fabio and Maree, 2016). The definition of a healthy organization is fundamental in this approach (De Smet et al., 2007; Grawitch and Ballard, 2016), and a healthy organization can be considered an organization whose culture, climate, and practices generate an environment that promotes employee health and safety as well as organizational effectiveness (Lowe, 2010).

### Hedonic and eudaimonic well-being

In terms of positive psychology (Seligman and Csikszentmihalyi, 2000), two aspects of well-being can be distinguished: hedonic well-being and eudaimonic well-being. Hedonic well-being (Watson et al., 1988) has life satisfaction as a cognitive evaluation component (Diener et al., 1985) and the dominance of positive emotions over negative emotions as an affective evaluation component (Watson et al., 1988).

Eudaimonic well-being refers to the full functioning of the individual (Ryan and Deci, 2001), life meaning, and purposefulness (Waterman et al., 2010). Flourishing refers to social and psychological prosperity and well-being (Diener et al., 2010) and is seen as perceived success in important areas such as relationships, self-esteem, sense of purpose, and optimism.

ISC, as a core of individual intrapreneurial resources, can therefore be regarded as an asset for workers in the twenty-first century, helping them create innovative solutions when confronted with constraints imposed by the organizational challenging environment. ISC can promote healthy people in terms of both hedonic and eudaimonic well-being (Di Fabio and Gori, 2016c) and consequently also healthy organizations.

### Aim and hypotheses

The aim of the study was to examine the relationship between intrapreneurial self-capital (ISC) and life satisfaction and flourishing controlling for the effects of personality traits.

The following two hypotheses were formulated.

H1: A positive relationship will emerge between ISC and life satisfaction controlling for the effects of personality traits.
H2: A positive relationship will emerge between ISC and flourishing controlling for the effects of personality traits.

## Materials and methods

### Participants

Two hundred and fifty eight Italian workers of different private and public organizations participated in the study. Regarding gender, the 59% of the participants were men and the 41% were women. The participants' ages ranged from 29 to 58 years (*M* = 45.59, *SD* = 9.92).

### Measures

#### Big five questionnaire (BFQ)

To evaluate personality traits, the Big Five Questionnaire (BFQ; Caprara et al., 1993) was used. The BFQ consists of 132 items with a response format on a 5-point Likert scale ranging from 1 = *Absolutely false* to 5 = *Absolutely true*. The questionnaire measures five personality traits. The Cronbach's alpha coefficients were 0.81 for Extraversion (example of item: “I think that I am an active and vigorous person”), 0.73 for Agreeableness (example of item: “I understand when people need my help”), 0.81 for Conscientiousness (example of item: “I tend to be very thoughtful”), 0.90 for Emotional stability (example of item: “I do not often feel tense”), and 0.75 for Openness (example of item: “I am always informed about what is happening in the world”).

#### Intrapreneurial self-capital scale (ISCS)

To evaluate intrapreneurial self-capital, the Intrapreneurial Self-Capital Scale (ISC; Di Fabio, 2014) was used. The ISCS consists of 28 items (e.g., “I am able to deal with most of my problems,” “I am able to improve the ideas produced by others,” “One of my goals in training is to learn as much as I can”) with a response format on a 5-point Likert scale ranging from 1 = *Strongly agree* to 5 = *Strongly disagree*. Cronbach's alpha coefficient was 0.84 (Di Fabio, 2014).

#### Satisfaction with life scale (SWLS)

The Satisfaction With Life Scale (SWLS, Diener et al., 1985) in the Italian version by Di Fabio and Gori (2015) was used to evaluate life satisfaction. The scale consists of five items (e.g., “I am satisfied with my life,” “The conditions of my life are excellent”) on a 7-point Likert scale ranging from 1 = *Strongly disagree* to 7 = *Strongly agree*. Cronbach's alpha coefficient was 0.85.

#### Flourishing scale (FS)

The Flourishing Scale (FS, Diener et al., 2010) in the Italian version by Di Fabio (2016) was used to evaluate flourishing. The FS consists of eight items with response options on a 6-point Likert scale ranging from 1 (*Strongly disagree*) to 7 (*Strongly agree*). Examples of items: “My social relationships are supportive and rewarding,” “I lead a purposeful and meaningful life,” “I am optimistic about my future.” The FS showed a unidimensional structure with good reliability in the present study (α = 0.88).

### Procedure and data analysis

The questionnaires were administered in groups by trained psychologists. The order of administration was counterbalanced to control the possible effects of a set presentation of the instruments.

The study assured to respondents anonymity and confidentiality. The questionnaire included a statement regarding the personal data treatment, in accordance with the Italian privacy law (Law Decree DL-196/2003). The workers authorized and approved the use of anonymous/collective data for possible future scientific publications. Because the data was collected anonymously and the research investigated psycho-social variables not adopting a medical perspective, ethical approval was not sought.

Descriptive statistics, Pearson's *r* correlations and hierarchical regressions were used. For the studied variables, gender differences were analyzed in distinct regressions for gender but no differences emerged. Therefore, only regressions for the entire sample are presented in the results session.

## Results

Means, standard deviations, and correlations between the BFQ, ISCS, SWLS, and FS are shown in Table 1.

**Table 1:** Means, standard deviations, and correlations between BFQ, ISCS, SWLS, and FS.

	***M***	***DS***	**1**	**2**	**3**	**4**	**5**	**6**	**7**	**8**
1. BFQ extraversion	77.06	13.30	–							
2. BFQ agreeableness	80.76	12.47	0.50^**^	–						
3. BFQ conscientiousness	81.72	12.80	0.48^**^	0.47^**^	–					
4. BFQ emotional stability	71.81	13.90	0.43^**^	0.41^**^	0.22^**^	–				
5. BFQ openness	83.52	12.98	0.53^**^	0.50^**^	0.40^**^	0.38^**^	–			
6. ISCS	103.00	14.57	0.31^**^	0.20^**^	0.18^**^	0.16^**^	0.28^**^	–		
7. SWLS	24.01	6.35	0.50^**^	0.45^**^	0.38^**^	0.39^**^	0.38^**^	0.44^**^	–	
8. FS	43.22	7.98	0.58^**^	0.50^**^	0.39^**^	0.40^**^	0.50^**^	0.52^**^	0.64^**^	–

*N = 258*.

** *p < 0.01. BFQ, Big Five Questionnaire; ISCS, Intrapreneurial Self-Capital Scale; SWLS, Satisfaction With Life Scale; FS, Flourishing Scale*.

Table 2 shows the results of two different hierarchical regression models, alternatively with life satisfaction and flourishing as the criterion measures and with personality traits at the first step and intrapreneurial self-capital at the second step.

**Table 2:** Hierarchical regression.

	**SWLS**	**FS**
	**β**	**β**
*Step 1*		
BFQ extraversion	0.24^**^	0.31^**^
BFQ agreeableness	0.18^*^	0.11
BFQ conscientiousness	0.12	0.01
BFQ emotional stability	0.17^*^	0.13^*^
BFQ openness	0.12	0.14^*^
*Step 2*	0.32^***^	0.37^***^
**ISCS**		
*R*^2^ step 1	0.31^***^	0.39^***^
*ΔR*^2^ step 2	0.09^***^	0.12^***^
*R*^2^ total	0.40^***^	0.51^***^

*The contributions of personality traits (first step) and intrapreneurial self-capital (second step) to life satisfaction (SWLS) and flourishing (FS)*.

*N = 258*.

* *p < 0.05*.

** *p < 0.01*.

*** *p < 0.001*.

*BFQ, Big Five Questionnaire; ISCS, Intrapreneurial Self-Capital Scale; SWLS, Satisfaction With Life Scale; FS, Flourishing Scale*.

At the first step, personality traits accounted for 31% of the variance in life satisfaction. At the second step, intrapreneurial self-capital added 9% of the incremental variance. The model overall accounted for 40% of the variance.

At the first step, personality traits accounted for 39% of the variance in flourishing. At the second step, intrapreneurial self-capital added 12% of the incremental variance. The model overall accounted for 51% of the variance.

## Discussion

The aim of the present study was to examine the relationship between intrapreneurial self-capital, life satisfaction and flourishing controlling for the effects of personality traits.

The first hypothesis was confirmed as intrapreneurial self-capital added significant incremental variance beyond that accounted for by personality traits in relation to life satisfaction. The present study showed that intrapreneurial self-capital helped create individual resources the participants could use to deal with twenty-first century challenges and thus also the challenges of everyday existence (Di Fabio, 2014) thereby contributing to greater hedonic well-being in terms of global satisfaction with their own careers and lives (Diener et al., 1985).

The second hypothesis, too, was confirmed as intrapreneurial self-capital added significant incremental variance beyond that accounted for by personality traits in relation to flourishing. These results supported the positive association of intrapreneurial self-capital (Di Fabio, 2014) with eudaimonic well-being (Di Fabio and Gori, 2016c) in terms of social and psychological prosperity and well-being in important areas such as relationships, self-esteem, presence of purpose, and optimism (Diener et al., 2010). It is interesting to note that ISC offered the biggest contribution to variance, especially in relation to flourishing, underlining the promising role of this core of intrapreneurial resources in promoting people as flourishing and resilient workers.

### Limitations

The study showed the positive contribution of ISC to life satisfaction and flourishing, yet a number of limitations can be mentioned. The sample was limited to a group of Italian workers who were not necessarily representative of all Italian workers. Future research should therefore extend the study of the relationship between these variables to participants from different geographical areas in Italy. Participants in other countries could also be included. Future research could also investigate ISC in relation to other aspects of hedonic and eudaimonic well-being (Watson et al., 1988) such as positive affect and meaning in life (Morgan and Farsides, 2009), subjective experiences of eudaimonia (Waterman et al., 2010), and existential fulfillment (Längle et al., 2003). Future research could also investigate ISC in relation to job satisfaction (Drydakis, 2017). Despite these limitations, the results add to the literature on the subject by underlining the contribution of ISC to life satisfaction and flourishing.

### Practical implications

If future research confirms the results of the present study, interventions could be introduced to enhance ISC thereby helping people face more successfully the unpredictable and changing environment in twenty-first century organizations. This would promote both individual and organizational well-being as seen from a primary prevention perspective in positive psychology (Hage et al., 2007; Di Fabio and Saklofske, 2014b; Di Fabio and Kenny, 2016b).

## Conclusions

In conclusion, ISC, as a core of individual intrapreneurial strengths, can be regarded as a key resource that workers can use to deal with the ongoing changes in the twenty-first century working environment (Di Fabio, 2014; Di Fabio and Gori, 2016a). The contribution of ISC to different aspects of workers' well-being highlights, from an organizational positive psychology perspective, the importance of enhancing individual resources (Di Fabio and Saklofske, 2014a,b; Di Fabio, 2015) to promote healthy businesses and healthy organizations (Boyatzis et al., 2002, 2015; Boyatzis and Saatcioglu, 2008; Boyatzis, 2009; Di Fabio and Blustein, 2016; Di Fabio and Kenny, 2016a,b; Di Fabio et al., 2016).

## Author contributions

ADF conceptualized the study, choose the theoretical framework and the measures. OB helped in the collection of the data. ADF and LP analyzed the data and wrote the methods and results. Then all authors wrote the paper together and read and revised the manuscript several times.

### Conflict of interest statement

The authors declare that the research was conducted in the absence of any commercial or financial relationships that could be construed as a potential conflict of interest.
